# 73. Interim Resistance Analysis of Long-Acting Lenacapavir in Treatment-Naïve People with HIV at 28 Weeks

**DOI:** 10.1093/ofid/ofab466.073

**Published:** 2021-12-04

**Authors:** Laurie VanderVeen, Nicolas Margot, Vidula Naik, Silvia Chang, Ross Martin, Hadas Dvory-Sobol, Martin Rhee, Christian Callebaut

**Affiliations:** Gilead Sciences, Foster City, California

## Abstract

**Background:**

Lenacapavir (LEN) is a first-in-class HIV-1 capsid (CA) inhibitor in clinical development for treatment and prevention of HIV-1 infection. CALIBRATE is an ongoing, phase 2 clinical study evaluating subcutaneous (SC) or oral LEN, in combination with other antiretrovirals, in treatment-naïve people with HIV-1. High rates of virologic success (HIV-1 RNA < 50 copies/mL) were achieved with LEN-based regimens by FDA Snapshot analysis at Week 28. Here, we present interim resistance analyses through Week 28.

**Methods:**

Participants were randomized (2:2:2:1) to treatment groups (TG) (Figure): SC LEN + oral daily emtricitabine/tenofovir alafenamide (F/TAF); at Week 28, participants switch F/TAF to oral TAF (TG-A) or bictegravir (B, BIC) (TG-B); oral daily LEN + F/TAF (TG-C), or oral daily B/F/TAF (TG-D). Genotypic analyses (population sequencing) of HIV-1 reverse transcriptase and integrase, and genotypic (deep sequencing)/phenotypic analyses for CA were performed at screening; genotypic and phenotypic analyses were conducted at confirmed virologic failure.

**Figure:**

CALIBRATE Study Design

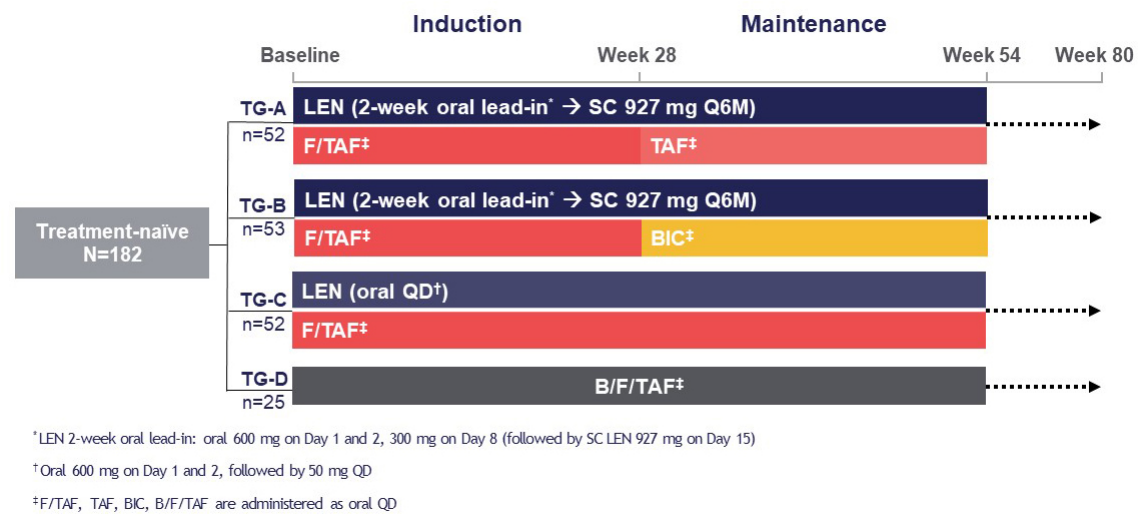

**Results:**

182 participants were randomized and dosed in TG-A to D (n=52, 53, 52, 25). Most participants had subtype B HIV-1 (92.9%). Sequence analysis of baseline samples found 65% of amino acid residues were conserved with < 1% variation across CA overall, and 55% of residues were fully conserved. No mutations were detected at 6 positions in CA associated with reduced susceptibility to LEN in vitro; residues were fully conserved at 5 positions (L56, M66, Q67, K70, N74), and < 2% variation was observed at 1 position (T107). Three participants met the criteria for resistance analysis: 2 participants resuppressed to < 50 copies/mL while continuing treatment. One participant on SC LEN + F/TAF developed emergent resistance to LEN (Q67H+K70R) and emtricitabine (M184M/I), followed by resuppression after starting dolutegravir, zidovudine + lamivudine, tenofovir disoproxil fumarate.

**Conclusion:**

Emergent resistance to LEN was uncommon in treatment-naïve participants receiving SC or oral LEN (0.6%, 1/157). These interim resistance findings support the ongoing evaluation of LEN for treatment and prevention of HIV.

**Disclosures:**

**Laurie VanderVeen, PhD**, **Gilead Sciences** (Employee, Shareholder) **Nicolas Margot, MA**, **Gilead Sciences** (Employee, Shareholder) **Vidula Naik, MSc**, **Gilead Sciences** (Employee, Shareholder) **Silvia Chang, Masters**, **Gilead Sciences, Inc** (Employee, Shareholder) **Ross Martin, PhD**, **Gilead Sciences, Inc** (Employee, Shareholder) **Hadas Dvory-Sobol, PhD**, **Gilead Sciences** (Employee, Shareholder) **Martin Rhee, MD**, **Gilead Sciences** (Employee, Shareholder) **Christian Callebaut, PhD**, **Gilead Sciences** (Employee, Shareholder)

